# MouBeAT: A New and Open Toolbox for Guided Analysis of Behavioral Tests in Mice

**DOI:** 10.3389/fnbeh.2018.00201

**Published:** 2018-09-07

**Authors:** Elísabet Bello-Arroyo, Hélio Roque, Alberto Marcos, Javier Orihuel, Alejandro Higuera-Matas, Manuel Desco, Valeria R. Caiolfa, Emilio Ambrosio, Enrique Lara-Pezzi, María Victoria Gómez-Gaviro

**Affiliations:** ^1^Centro Nacional de Investigaciones Cardiovasculares (CNIC), Madrid, Spain; ^2^Unidad de Microscopía e Imagen Dinámica, Centro Nacional de Investigaciones Cardiovasculares (CNIC), Madrid, Spain; ^3^Departamento de Psicobiología, Universidad Nacional de Educación a Distancia (UNED), Madrid, Spain; ^4^Departamento de Medicina y Cirugía Experimental, Instituto de Investigación Sanitaria Gregorio Marañón, Madrid, Spain; ^5^Departamento de Bioingeniería e Ingeniería Aeroespacial, Universidad Carlos III de Madrid, Getafe, Spain; ^6^Unidad de Imagen Avanzada, Centro Nacional de Investigaciones Cardiovasculares (CNIC), Madrid, Spain; ^7^Centro de Investigación Biomédica en Red de Salud Mental, CIBERSAM, Madrid, Spain; ^8^Ospedale San Raffaele, Centro di Immagine Sperimentale, Milan, Italy; ^9^Centro de Investigación Biomédica en Red de Enfermedades Cardiovasculares (CIBERCV), Madrid, Spain; ^10^National Heart and Lung Institute, Faculty of Medicine, Imperial College London, London, United Kingdom

**Keywords:** behavioral tests, rodents, open source, ImageJ, semi-automated analysis

## Abstract

Animal behavioral tests are essential to understand the bases of neurologic and psychological disorders, which can be evaluated by different methodological and experimental models. However, the quantification of behavioral tests results is limited by the considerable amount of time needed for manual evaluation and the high costs of automated analysis software. To overcome these limitations, we describe here a new, open source toolbox for ImageJ, called Mouse Behavioral Analysis Toolbox (MouBeAT), designed to analyze different behavioral tests in rodents semi-automatically. These tests include Open Field (OF), Elevated Plus Maze (EPM), Y-maze (YM) test and Morris Water Maze (MWM). MouBeAT showed a high correlation with manual evaluation in all the parameters analyzed for all the behavioral tests, reinforcing its value as an accurate analysis tool. This new tool is freely available online.

## Introduction

Over the past several decades, the need to improve our understanding of human neuropsychiatric disorders has driven the development of new animals models and methodologies that facilitate drug testing and the identification of new disease mechanisms (Nestler and Hyman, 2010). Rodents are the most commonly used animals in experimental research, including that oriented towards neurological diseases (Van Meer and Raber, 2005). Different behavioral tests have been designed to investigate different aspects of mouse behavior, including anxiety, cognition, spatial memory and learning. However, manual quantification of the results of these tests are susceptible to inter-observer variability and are time consuming, whereas automated systems are often unaffordable. Therefore, new affordable automated tools are needed to enable accurate analysis of mouse behavior.

A number of commercial automated programs for the analysis of behavior in rodents have been developed, including Ethovision (Noldus et al., 2001), Top Scan (CleverSys Inc.), Smart Video Tracking Software (Panlab Harvard Apparatus), VideoTrack (View Point, Behavior Technology) and AnyMaze (Stoelting Co., Wood Dale, IL, USA). Most of them are sold as a hardware and software package, making these options unaffordable for many laboratories. Some of the publicly available software options need a MATLAB^®^ license (Aragão et al., 2011), which results in an increased cost, take much longer to run or are limited to the operating systems or tasks with which they can be used (Patel et al., 2014). Other open source options, are very directed to tracking (Freeman and Ambady, 2010; Crispim Junior et al., 2012; Kabra et al., 2013; Pérez-Escudero et al., 2014; Ben-Shaul, 2017) or detecting grooming trajectories (Reeves et al., 2016). There are a few open source programs to evaluate multiple behavioral test in mice. However, those are designed for online analysis, they focus on only one specific test and they provide information about movement parameters without taking into account other variable such as the number of entries to different regions of interest (Shoji et al., 2014; Samson et al., 2015).

Here, we present Mouse Behavioral Analysis Toolbox (MouBeAT), a new open-source set of tools to analyze semi-automatically different behavioral tests in rodents. It is a user-friendly program based on the widely used and available image analysis software ImageJ^1^. In addition, like ImageJ, it is platform independent. We designed MouBeAT to process videos in a rapid manner, having no special requirements for their recording. MouBeAT shows high correlation with manual analysis and can be used for the analysis of several behavioral tests, including Open Field (OF), Elevated Plus Maze (EPM), Y-Maze (YM) and Morris Water Maze (MWM).

## Materials and Methods

### Animals

Ten adult male C57BL/6J mice at 20 weeks of age were purchased from Charles River (Sulzfeld, Germany) and used for the different behavioral tests. All experiments were approved by the local Ethics Committee at the Universidad Nacional de Educación a Distancia (UNED). All animals were maintained and handled according to European Union Laboratory Animal Care Rules (86/609/EEC Directive). During behavioral experiments, 2–4 mice were housed per cage under a normal 12:12 h light/dark cycle (lights on at 8:00 h) in standard laboratory conditions (22°C, 55% humidity, food and water *ad libitum*). All behavioral tests were conducted daily in the light cycle between 13:00 h and 18:00 h and following the same order with animals every day so that the time lapse between mice was maintained constant. Animals were allowed to adapt to the specific requirements of each particular device for a few minutes before the test itself. Mice were placed in the experimental room at least 30 min before test for habituation. The appliances were cleaned between each mouse using 70% ethanol.

### Video Recording

All tests were recorded in a zenithal view with a color video CCD camera (Model CP-720) placed at the center of each apparatus. The camera was coupled to a varifocal lens AVENIR cctvlens 3.5–8.0 mm F1.4 and videos were acquired in an AVI file format. The recording time was adjusted as needed for each particular test, ranging from 1 min to 5 min. The time needed to perform the analysis depends on the number of frames of each individual video, computer characteristics and user handling. Our analysis lasted ~5 min. Our videos had no more than 9,000 frames. The computer used to analyze the videos had the following characteristics: Processor: Intel^®^ Core™ i7-4770 CPU @ 3.4 GHz (8 CPUs), 3.4 GHz; Memory: 32 GB RAM; Operating system: Windows 7 Professional 64-bit (6.1, Build 7601). Measured parameters are indicated below for each specific test. MouBeAT quantifies displacement, time spent in different regions and average velocity of every mouse in all tests. Moreover, it extracts the pathway followed by each mouse with two track maps (line and heat map).

### Open Field Test

A square box (38 × 38 × 40 cm) made of beige Plexiglas plastic and illuminated in the center above it (30 lx) was used for the OF test. The floor of the box was divided into 5 × 5 quadrants (each individual quadrant being 7.6 cm long × 7.6 cm wide, making a field of 38 × 38 cm). The central area was defined as the central 3 × 3 quadrant region (22.8 × 22.8 cm). Mice were placed individually in the center of the area and allowed to freely explore it for 5 min while being recorded (Van Meer and Raber, 2005). MouBeAT quantifies the distance and time spent in the central and peripheral areas, number of entries to center region and freezing time in each area. Freezing time was defined as the absence of all movement. An entry into an area was counted when the four legs of the mouse (for manual quantification) or at least 70% of the mouse body (for MouBeAT) had completely entered the area.

### Elevated Plus Maze

The maze consisted of a plus-shaped device made of white plastic with two open and two closed arms (25 cm long × 7 cm width × 24.5 cm high walls of closed arms) elevated 40 cm from the ground. Mice were individually placed in the central area (8 × 8 cm, 30 lx) of the maze and allowed to explore for 5 min (An et al., 2011). The number of explorations over the edge defined as events where the mouse extends its head out of the edge of the open arms to look at the environment, also called “head-dips,” number of entries in closed and open arms and time spent in each arm were determined. An entry into a region was considered when the four legs of the mouse (for manual quantification) or 80% of the mouse body (for MouBeAT) had crossed into the open/closed arms or into the center region.

### Y-Maze Test

Mice were individually placed in the center of a Y-shaped maze (30 lx) with three light-gray plastic arms (7 cm wide, 35 cm long and with 15 cm high walls) at a 120° angle from each other, and were allowed to explore the three arms of the maze freely for 5 min. Mice were recorded to determine the number, duration and order of the visits to each arm. In order to calculate the percentage of alternation between maze arms, we measured three consecutive entries in different arms divided by the total of entries minus 2 (Wolf et al., 2016). A region entry was established when the four legs of the mouse (for manual quantification) or 90% of the mouse body (for MouBeAT) had crossed its boundaries. Exit was considered when half of the body had moved out (for manual quantification) or when 90% of the mouse body is out of the region (for MouBeAT).

### Morris Water Maze Test

The MWM test was conducted in a circular pool (125 cm diameter) with four external maze cues in the room walls. The protocol consisted of four trials per day and mouse during six consecutive days (Bromley-Brits et al., 2011). In the first day, mice were individually placed for 1 min in the pool filled with water, in which there was a visible platform (10 cm diameter) and a flag indicating the escape. Pool temperature was maintained at 25 ± 0.5°C. The following 4 days, the water was colored with white non-toxic tempera paint (Jovi^®^) so that mice could not see the hidden platform. In order to reach it, they had to follow external maze cues. On the last day, the platform was removed and the mice were recorded for 1 min to see latency and the pathway to find platform. Furthermore, the distance and time spent in each quadrant and the time spent close to pool wall was quantified (Vorhees and Williams, 2006) during the probe trial. A mouse entry or exit in a quadrant was counted when the four legs of the mouse (for manual quantification) or the center of mass of the mouse (for MouBeAT) were inside the quadrant.

### Video Importing and Pre-processing Tools in MouBeAT

Videos were acquired in AVI format at 25 frames per second (fps). To import videos to ImageJ, the FFMPEG importer was used^2^. In addition, the Toolbox provides two pre-importing functions to convert compressed AVI files to uncompressed AVI files, either individually or in batch. These functions require the open source software FFmpeg to be available in the user’s computer. In addition, these files can be converted into an image stack file of Tiff format, which can be readily opened with ImageJ. The pre-importing individual function also allows for files to be cropped, removing unnecessary parts and reducing file size. Once imported, preprocessing is dependent on user choice. All tools allow a Gaussian Blur filter to be applied, with the user able to choose the radius of decay to *e*^−1/2^ in pixels. In addition, all tools allow for background subtraction and correction. An average projection of the stack is created, which is subsequently used to produce a difference stack of the original movie.

### MouBeAT Detection and Tracking

Mouse detection was performed by imposing a “Minimum” auto threshold to the processed stack (Prewitt and Mendelsohn, 1966). Upon this, the user has the possibility of manually adjusting the threshold. The threshold regions are then converted into region of interests (ROIs) which are used for all subsequent analysis. ROIs were only considered if above a predetermined area (set in the Toolbox preferences—see below). In addition, ROI borders were smoothed to eliminate rough edges, by applying one cycle of expansion/erosion of the selection. The displacement in between frames was calculated using the Euclidean distance formula for the centroid coordinates (*x*, *y*) of each frame (*i*): $\text{Δ}{\text{d}}_{\left(i,i+1\right)}=\sqrt{{\left(x_{i+1}-x_{i}\right)}^{2}+{\left(y_{i+1}-y_{i}\right)}^{2}}$. When Δ*d* was smaller than a settable variable (in Preferences), to accommodate for small variances in threshold detection between frames, the mouse was considered not to have moved. Speed was calculated for each frame time interval (*t*): $s=\frac{\text{Δ}d}{\text{Δ}t}$. To determine the mouse position inside regions, the perimeter coordinates of ROIs were examined for location in each frame and assigned to a region, with a set fraction determining where the mouse was located.

### MouBeAT Design and Operation

**Figure 1:**
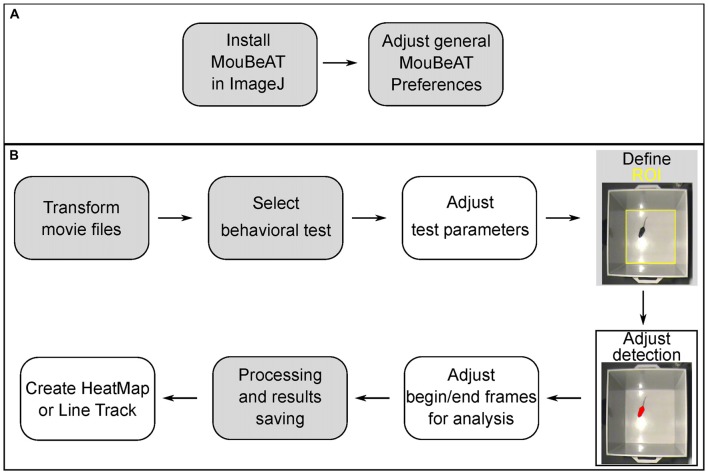
Workflow of Mouse Behavioral Analysis Toolbox (MouBeAT) analysis. **(A)** Onetime steps of installation and adjustment of general preferences. **(B)** Main steps for specific tests. Gray filled boxes indicate mandatory steps while empty boxes indicate optional steps. Yellow region of interest (ROI) represent areas created by the user to defined regions of the maze during the workflow in this and all subsequent figures.

The Toolbox is totally written in ImageJ Macro Language (Schneider et al., 2012). MouBeAT functions to convert AVI files are dependent on the file ffmpeg.exe to be present in the ImageJ macro\toolset folder. This is the only prerequisite for MouBeAT Toolbox. All other functions work with the basic ImageJ install. MouBeAT operation starts with setting the preferences, from the Preferences Menu (Figure 1A). Here, general and specific algorithm preferences can be set. An option to take preference defaults, which we set from our data, is available. After this, the user can perform the desired Behavioral Analysis by selecting it from the dropdown menu (Figure 1B). This will open a specific user interface with settings to allow the determination of the pixel size of the mazes and other processing options. The software then guides the user through the subsequent steps, according to the chosen options. At the end of the analysis, two EXCEL files are generated and stored, one with the frame-by-frame analysis and another one with the overall results. In addition, the determined ROIs of the analysis are also saved. The analysis also allows for the creation of a heat map and a trajectory map of the mouse. Finally, a file containing preferences, and analysis parameters is saved, which allows for the analysis to be repeated later with the same parameters, or for the heat map and/or trajectory map to be recreated. Further details of use are included in the provided MouBeAT user guide (see Supplementary Material).

### Statistical Analysis and Validation

GraphPad Prism 7 was used to perform all statistical analyses and graphs. Correlations between manual and MouBeAT measurements were analyzed by establishing manual observations as the gold standard. CPP-OF V.19 Tiselius S.L software was used to help with manual validation (Santos-Toscano et al., 2016). Ten wild type mice were used for all tests and 10 videos were recorded per task to validate the toolbox. Depending on whether the results obtained for each sample followed a Gaussian distribution or not, Pearson’s or Spearman correlation coefficients (r) between two methods were calculated to compare the results, respectively.

## Results

### Anxiety-Related Behavior Tests

To determine the reliability of MouBeAT, we compared the results obtained with this tool to those obtained with manual analysis in different behavioral tests commonly used in the neuropsychological field, including OF, EPM, YM and MWM (Table 1). We first tested the program on anxiety-related behavior tests.

**Table 1:** Automated parameters measured by Mouse Behavioral Analysis Toolbox (MouBeAT) in five behavioral tests.

Behavioral test	Automated parameters calculated by MouBeAT
Open Field	Distance traveled and time spent in center and outer region. Number of entries in center. Freezing time in center and outer region. Speed average.
Elevated Plus Maze	Distance traveled and time spent in central area, closed and open arms. Number of entries in closed and open arms. Speed Average. Times of over the edge exploration.
Y-maze	Distance traveled. Number of visits, time spent and time to first visit to center, left and right arm. Speed average. Order of arms entry. Percentage of alternation = different triplets/total triplets.
Morris Water Maze	Distance traveled and time spent in every quadrant. Time close to pool wall (10 cm). Speed Average. Time to find platform.

**Figure 2:**
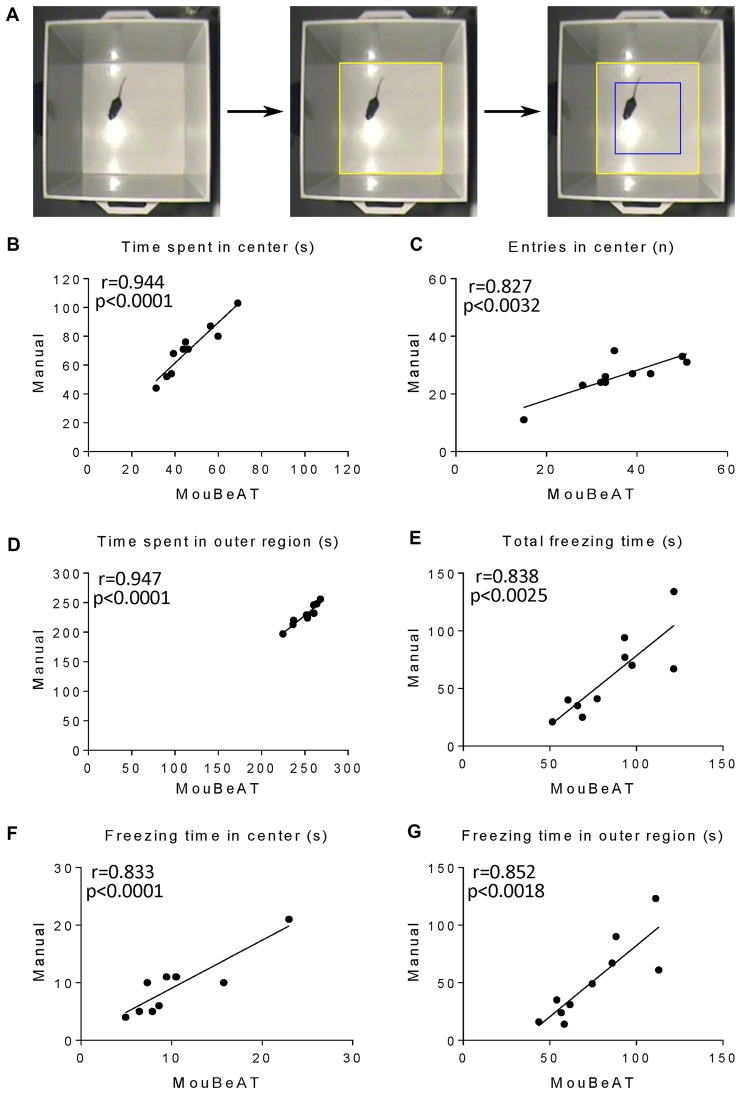
Correlation of the measured variables in the open field (OF) test between MouBeAT and manual assessment. The OF test **(A)** was performed using 10 C57BL/6J male mice. Yellow ROI defines the base of the cube while blue ROI indicates the center region calculated by the software. Each dot represents time spent in the center **(B)**, number of entries into the center **(C)**, time spent in outer region **(D)**, total freezing time **(E)**, freezing time in the center **(F)** and freezing time in the outer region **(G)** for each mouse. In this and all subsequent Figures Pearson’s correlation coefficients were calculated for all variable except in cases where data followed a non-parametric distribution, in which case the Spearman correlation coefficient was used. Individual *p* values are displayed in the Figures. For **(F)** the Spearman correlation coefficient was used.

**Figure 3:**
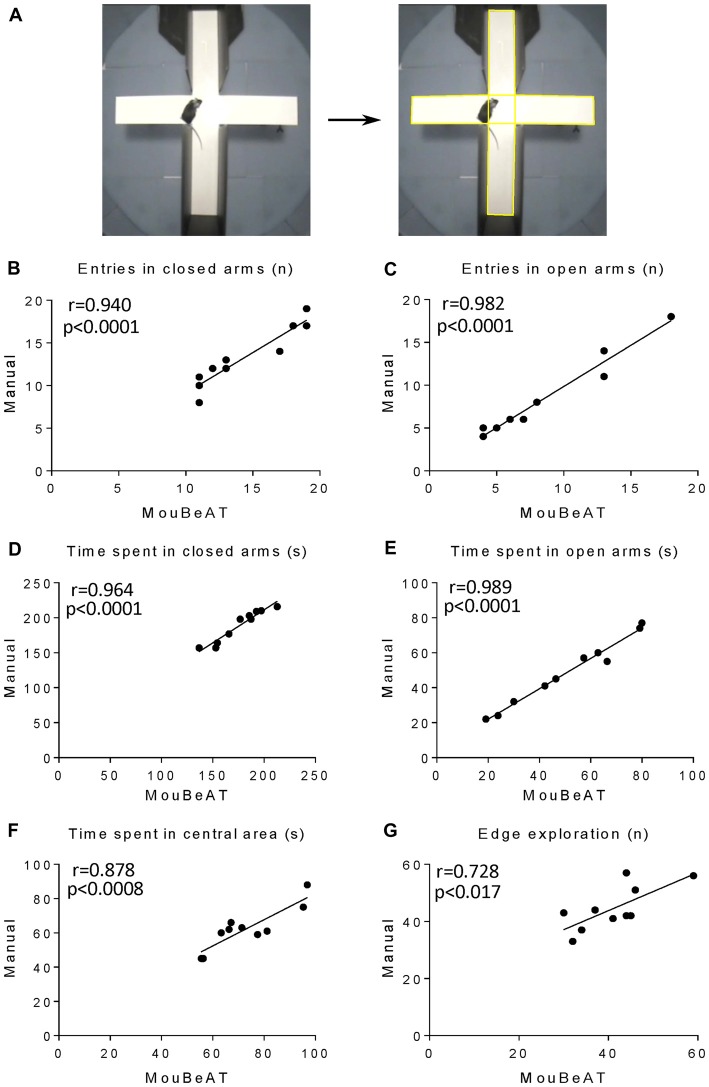
Correlation of results for the Elevated Plus Maze (EPM) obtained by MouBeAT and manual observation.The EPM test **(A)** was performed on 10 C57BL/6J male mice. Yellow ROIs defines the central region and edges of the maze. Each dot represents the number of entries in closed **(B)** and open **(C)** arms, time spent in closed **(D)** and open **(E)** arms, time spent in central area **(F)** and times of over the edge exploration **(G)** for each mouse.

The OF test (Figure 2A) is an assay used to study anxiety-like behaviors in rodents with detection of locomotor activity and exploration (Walsh and Cummins, 1976). Long periods spent in the center of the device and a high number of exits/entries to the center square are related with low levels of anxiety (Supplementary Video S1). In contrast, reduced movement of the mouse indicates increased levels of anxiety. As shown in Figures 2B–D, MouBeAT showed a strong correlation with the manual analysis for the time spent in the center (*r* = 0.944; *p* < 0.0001), the number of entries in the center (*r* = 0.827; *p* < 0.0032, see Table 2 for details on regression slopes) and the time spent in the outer region (*r* = 0.947; *p* < 0.0001). Similarly, both approaches showed high correlation for the overall freezing duration (Figure 2E; *r* = 0.838; *p* < 0.0025), freezing time in the center (Figure 2F; *r* = 0.833; *p* < 0.0001) and freezing time in the outer region (Figure 2G; *r* = 0.852; *p* < 0.0018). The results also showed that MouBeAT slightly overestimated the entries in center and freezing times compared to the manual assessment (see “Discussion” section). To investigate further the reliability of MouBeAT for the assessment of anxiety-related behavior, we tested it in the EPM test (Figure 3A; Supplementary Video S2), in which time spent and number of entries in open and closed spaces are evaluated (Walf and Frye, 2007). As illustrated in Figures 3B,C, MouBeAT determined the number of entries in the closed and open arms with great accuracy, showing a strong correlation with the data obtained using the manual approach (*r* = 0.940; *p* < 0.0001 and *r* = 0.982; *p* < 0.0001, respectively). The time spent in closed and open arms, and the time spent in central area also showed good correlation with the manual method (Figures 3D–F; *r* = 0.964; *p* < 0.0001, *r* = 0.989; *p* < 0.0001, *r* = 0.878; *p* < 0.0008 respectively). Finally, the number of times that the mouse explored the edges of the board was also determined by MouBeAT (Figure 3G; *r* = 0.728; *p* < 0.017), although it showed a slight underestimation compared to manual scoring (see “Discussion” section). Together, these results demonstrate that MouBeAT is a reliable tool for the quantification of anxiety-related behavior in mouse.

**Table 2:** Details of the regression slopes.

Test	Figure	Variable measured	Slope (a ± SE)
Open field	2B	Time spent in center (s)	1.41 ± 0.173
	2C	Entries in center (n)	0.517 ± 0.124
	2D	Time spent in outer region (s)	1.19 ± 0.143
	2E	Total freezing time (s)	1.21 ± 0.279
	2F	Freezing time in center (s)	0.833 ± 0.153
	2G	Freezing time in outer regions (s)	1.23 ± 0.268
Elevated Plus	3B	Entries in closed arms (n)	0.947 ± 0.122
Maze	3C	Entries in open arms (n)	0.962 ± 0.0645
	3D	Time spent in closed arms (s)	0.945 ± 0.0919
	3E	Time spent in open arms (s)	0.869 ± 0.0461
	3F	Time spent in central area (s)	0.765 ± 0.148
	3G	Edge exploration (s)	0.668 ± 0.222
Y-maze	4B	Entries in center arm (n)	0.988 ± 0.031
	4C	Entries in left arm (n)	1.07 ± 0.0523
	4D	Entries in right arm (n)	0.911 ± 0.0556
	4E	Time spent in center arm (s)	1.03 ± 0.0339
	4F	Time spent in left arm (s)	1.07 ± 0.147
	4G	Time spent in right arm (s)	0.97 ± 0.0778
	5A	Alternation (%)	0.593 ± 0.253
	5B	Time to first center arm visit (s)	1.02 ± 0.0321
	5C	Time to first left arm visit (s)	0.993 ± 0.0268
	5D	Time to first right arm visit (s)	1.01 ± 0.0193
Morris Water	6B	Time to find platform (s)	0.985 ± 0.00426
Maze	6C	Time in Q1 (s)	1.01 ± 0.0212
	6D	Time in Q2 (s)	1.07 ± 0.038
	6E	Time in Q3 (s)	0.966 ± 0.0907
	6F	Time in Q4 (s)	1.1 ± 0.0856
	6G	Time closed to pool wall (s)	0.723 ± 0.0471

### Working Memory Test

**Figure 4:**
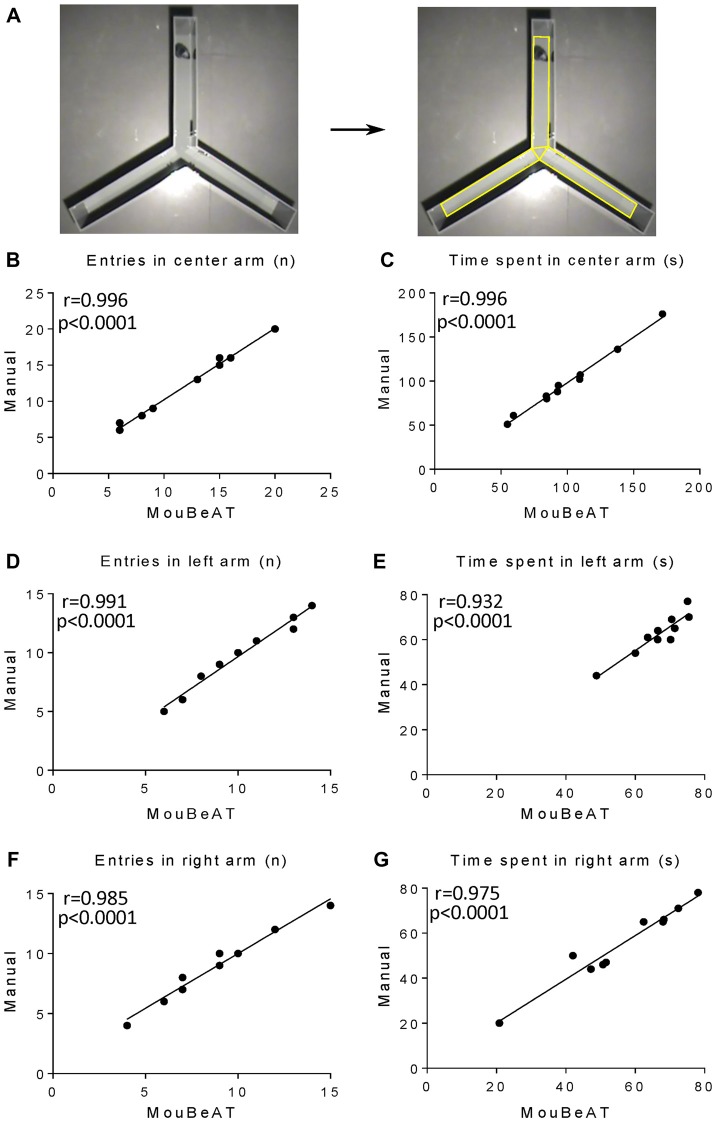
Correlation between the results obtained with MouBeAT and those from manual assessment for the measured variables in the Y-maze (YM) test. The YM test **(A)** was performed using 10 C57BL/6J male mice. Yellow ROI defines the edges and central triangle of the maze. Each dot represents the number of entries into the center **(B)**, left **(D)** and right arms **(F)**, and the time spent in the center **(C)**, left **(E)** and right **(G)** arms for each mouse.

**Figure 5:**
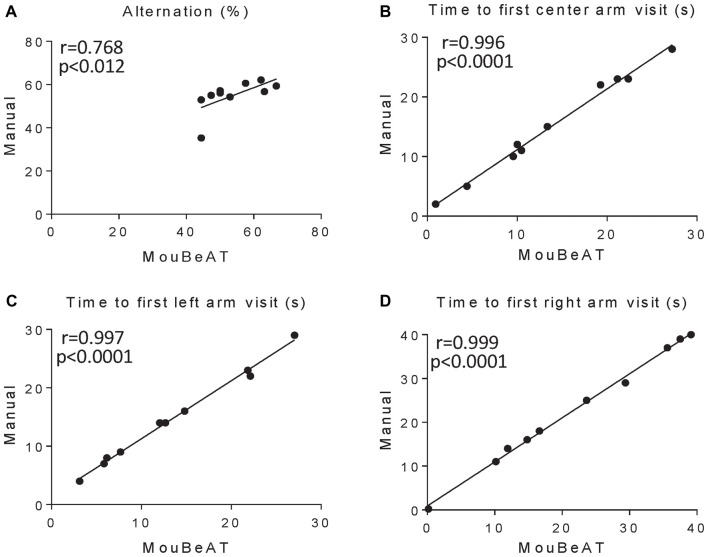
Correlation of arm alternation and time to visit each arm in the YM test between MouBeAT and manual observation. The YM test was performed using 10 C57BL/6J male mice. Each dot represents the percentage of alternation between arms **(A)**, and the time it took each mouse to visit the center **(B)**, left **(C)** and right **(D)** arms for the first time. For **(A)** the Spearman correlation coefficient was used.

To determine the suitability of MouBeAT to measure cognitive deficits and exploratory behavior, we used the YM test (Figure 4A, Supplementary Video S3). In this test, mice show a natural exploratory tendency to enter a new arm rather than to return to one previously visited. We first determined whether MouBeAT could accurately quantify the number of times that a mouse entered each of the three arms of the maze and the time spent in each one. As shown in Figure 4, MouBeAT showed a very strong correlation with the manual assessment of the number of entries and the time spent in the center arm (*r* = 0.996 and *r* = 0.996, respectively; Figures 4B,C). Similarly strong correlations were found for the number of entries into the left and right arm of the maze and the time spent in each one (*r* = 0.991, *r* = 0.985 for entries in left and right arms, respectively; *r* = 0.932, *r* = 0.975 for time spent in left and right arms, respectively; Figures 4D–G). All parameters measured in Figure 4 have a *p*-value < 0.0001. We next quantified the spontaneous alternation in exploration using the order of arm entries (Wahl et al., 2017). In this case, the results were skewed due to one animal having much less alternation compared to others (Figure 5A). In addition, MouBeAT showed a slight overestimation compared to the manual assessment (*r* = 0.768; *p* < 0.012; Figure 5A).

To further assess exploration behavior and spatial working memory, we also determined the time the mice took to enter each arm (Typlt et al., 2013; Peng et al., 2016). We found very high correlation between MouBeAT and manual quantification for latency to enter to all three arms of the maze (*r* = 0.996, *r* = 0.997, *r* = 0.999 for the center, left and right arms, respectively, *p* < 0.0001; Figures 5B–D), reinforcing the reliability of this tool. Of note, we found that placing the mouse initially in one arm of the maze, instead of the center of the device, allowed sufficient time for the researcher to remove the hand before the mouse entered any other arm, thereby avoiding missing an entry.

### Spatial Learning Test

**Figure 6:**
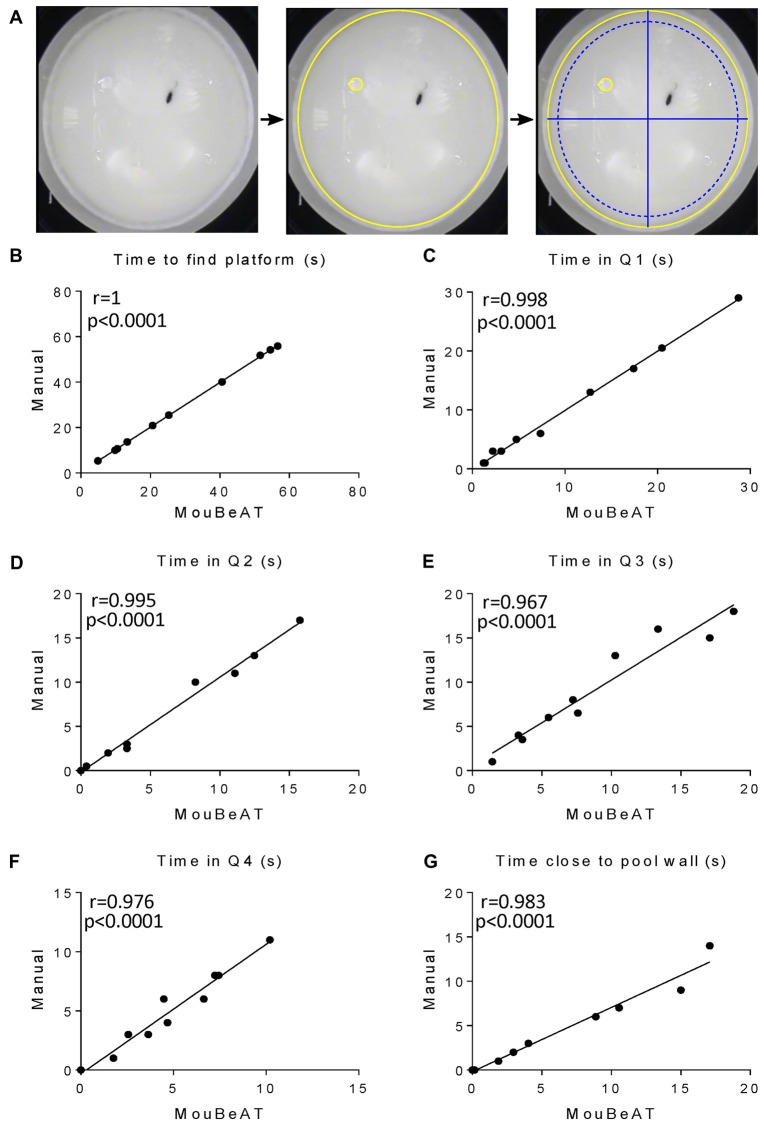
Correlation between MouBeAT and manual assessment of different parameters in the Morris Water Maze (MWM) test. The MWM test **(A)** was performed on 10 C57BL/6J male mice. Yellow ROI defines the edges and the platform regions, while blue ROIs define the quadrants and the region close to the pool wall, calculated by the software. Each dot represents the time needed by the mouse to find the platform **(B)**, time in quadrants 1–4 **(C–F)** and the time spent close to the pool wall **(G)** for each mouse.

To evaluate spatial learning, we used the MWM (Figure 6A), measuring the time that each mouse needed to find the platform, the time spent in each of the four quadrants and the time spent nearer the pool wall (Supplementary Video S4). Pearson’s correlation coefficient between manual and MouBeAT measurement of the time needed to find the platform approached *r* = 1 (Figure 6B). Moreover, MouBeAT was able to accurately determine whether a mouse did not find the platform in the allocated time, indicating it as “Did not find platform” in the results file (data not shown).

MouBeAT also accurately quantified the time spent by the mouse in each of the quadrants, with coefficients ranging between 0.967 and 0.998 (Figures 6C–F; Table 2). Finally, we found a very strong correlation between both methods in the quantification of the time spent by the mouse in the 10 cm closest to the pool wall (*r* = 0.983; Figure 6G). All parameters measured in Figure 6 have a *p*-value < 0.0001.

## Discussion

According to the World Health Organization (WHO), mental disorders are a major cause of disability worldwide. The need for new treatments and diagnostic tools is, therefore, urgent. The development of new therapeutic options relies on the use of animal models in which the effectiveness of the treatment can be measured accurately and efficiently. A battery of behavioral tests have been developed in mice over the last decades to increase our understanding of these diseases and to test the efficacy of new treatments (Chan-Palay et al., 1984; Hamann et al., 2002; Lalonde, 2002; Walf and Frye, 2007; Possin et al., 2016; Walz et al., 2016). However, these tests have limitations. Not least among these is the tendency to subjectivity between laboratories in the manual quantification of behavior patterns. In addition, manual scoring is considerably time consuming, since each video has to be played individually in its full length, often more than once, to accurately measure the different parameters. For these reasons, manual quantification began to be replaced by automated analysis. Currently, there are a number of resources available. However, these options are expensive (e.g., Ethovision; Noldus et al., 2001; Aragão et al., 2011) or are limited to the evaluation of a few number of parameters (e.g., only measuring locomotion activities (Tort et al., 2006; Freeman and Ambady, 2010; Crispim Junior et al., 2012; Kabra et al., 2013; Pérez-Escudero et al., 2014; Samson et al., 2015; Ben-Shaul, 2017; Tungtur et al., 2017). Here, we show that MouBeAT is not only an excellent free tracking method for different tasks but also a very specialized toolbox capable of assessing several variables required to evaluate multiple behavior tests. In addition, although it is originally developed for mouse behavioral analysis, it has the potential to be used for other non-rodent species. Results achieved with MouBeAT showed high correlation with those obtained by manual scoring.

In order to assess MouBeAT capabilities, and being unable to use other automated analysis, we restricted our comparison to manual scoring. MouBeAT has also some limitations associated with uneven illumination, which can cause shadow regions where the animal is not distinguishable from the background, leading to inaccurate quantification. In addition, it is not capable of detecting rearing, grooming or defecation. We found that MouBeAT presents slight differences to manual scoring in variables related with frontier crossing and freezing times. In the first case, due to the rigid criteria used by our software to determine when frontier is crossed, MouBeAT either over or underestimates the results compared to manual scoring. We believe this not to be a failure but a strength, since the criteria is not subjective to user interpretation as it happens with manual scoring. In the second case, we found that MouBeAT tends to overestimate freezing times. This we found to be a limitation of MouBeAT that can only detect if the animal is stopped and is unable to visualize finer details like head nodding or moving ears.

Despite these limitations, MouBeAT yields reliable results in a much shorter time than that needed for manual scoring. It is freely available, based on ImageJ, and runs on major operating system, including Linux, Windows and MacOS. In addition, the fact the MouBeAT was developed in a readily accessible, well documented and easily available coding language, allows for a speedy adaptation and extension of the toolbox for new applications, by users with an intermediate level of understanding of ImageJ macro language. The toolbox was designed to work with both white and black mice in black and white backgrounds, respectively, but since it can detect static vs. non-static elements, it has the possibility of working with variable backgrounds (something we did not test ourselves). We show here that this new automated toolbox is an excellent option to analyze common behavioral tests in rodents, such as OF, EPM, YM and MWM.

## Data Availability

The datasets generated for this study can be downloaded in the GitHub repository (https://github.com/helioalexandre/MouBeAT) or are included in the manuscript and Supplementary Material.

## Author Contributions

EB-A, EL-P and MVG-G: conceptualization. AH-M, EA, VC, MD, EL-P and MVG-G: supervision. EB-A, HR, AM, JO, AH-M, EA, VC, EL-P and MVG-G: methodology, data interpretation, review and editing and discussion. EB-A: experiment performance and data acquisition. EB-A, AM and EA: formal analysis. EB-A and AM: validation. HR and VC: software. EB-A, HR, EL-P and MVG-G: writing manuscript.

## Conflict of Interest Statement

The authors declare that the research was conducted in the absence of any commercial or financial relationships that could be construed as a potential conflict of interest.

## Footnotes

^1^https://imagej.nih.gov/ij/

^2^http://www.ffmpeg.org

## Supplementary Material

Further Supplementary Material for this article can be found at GitHub repository https://github.com/helioalexandre/MouBeAT. It contains a detailed PDF user guide for MouBeAT, source code (MouBeAT.ijm) and four example videos available to test all behavioral tasks. The avi files included are: Video S1 (OF test), Video S2 (EPM), Video S3 (YM) and Video S4 (MWM).
