# Supplementary material for: MouBeAT: A New and Open Toolbox for Guided Analysis of Behavioral Tests in Mice
Source: Front Behav Neurosci. 2018 Sep 7;12:201. doi: 10.3389/fnbeh.2018.00201 (PMC6137138; doi:10.3389/fnbeh.2018.00201)
Supplement: Supplementary file 1 [file Data_Sheet_1.PDF]

Mouse Behavioral Analysis Toolbox Manual

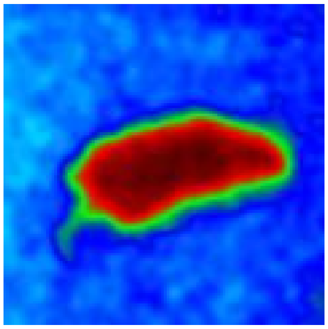

Contents

Getting Started 1
Preferences dialog 1
Transforming your video into a TIFF stack 3
Process Video File 3
Batch Process Video Files 3
Open Field 4
Elevated Plus Maze 6
Morris Water Maze 7
Y-maze 7
Open Previous Analysis File 8
Ouput Files 8
Known problems and workarounds 9

Getting Started

The first step to run the toolbox is to install it (plugin>macros>install) and it appears in the menu toolbar. If you download the files to the macro toolbox in ImageJ/FIJI, then the toolbox should be available on the list that you get by pressing the ImageJ/FIJI toolbar (at the very right of the menu toolbar). ImageJ/FIJI can be download from https://imagej.net/Fiji/Downloads.

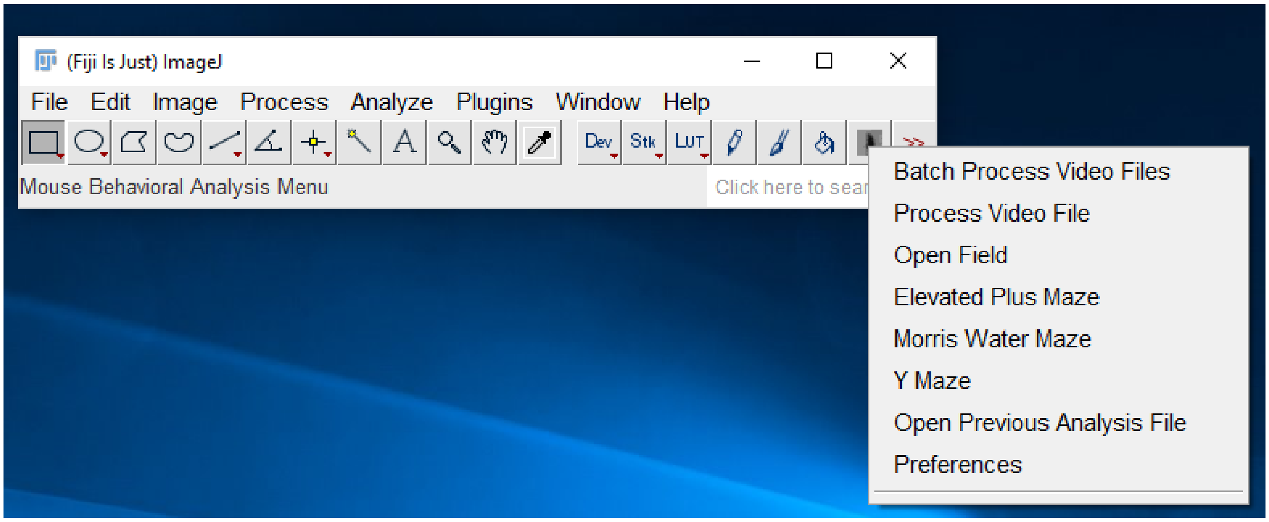

Figure 1. MouBeAT drop-down menu options

Preferences dialog

The preferences are normally asked for at the beginning of each specific process, but filling them here allows you to quickly jump through the dialog box. The preferences are organized into General Preferences and Test Specific. If you have no idea about what the preferences mean, just select the last checkbox which will fill the preferences with values that have worked well for our test movies. After this, for the values to take effect you will have to restart Image/FIJI and call the macro again.

General preferences

Units or work – this is required to put the correct units on the measurements. This does not affect the calculations but affects correctness.

Gaussian blur sigma value – sigma value to apply to the movie. This noise reduction is normally done at the beginning of processing to smooth out noise in the movie. A value of 0 means that no filter will be applied. This will have impact in the calculations!

Minimum displacement to consider for a moving mouse (units) – the movement of a mouse is calculated with relation to the displacement of its centroid from frame (n) to frame (n+1). Randomness normally means that the centroid will move minimally from frame to frame. This value sets the minimal threshold to consider that the mouse did move. Insert this value in true units (eg. centimeters).

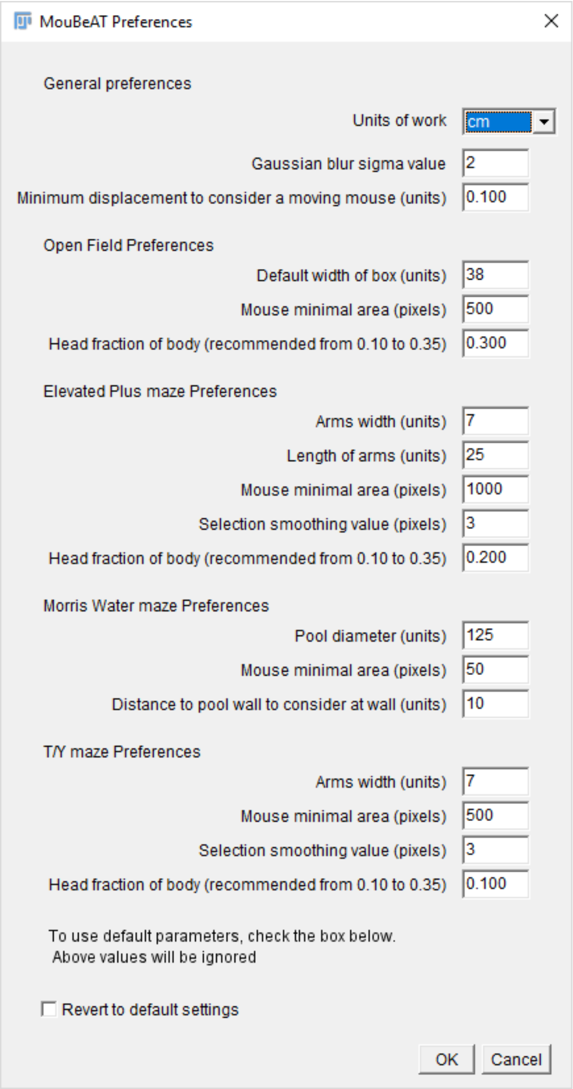

Figure 2. MouBeAT preferences dialog.

Preferences present in all analysis

Mouse minimal area (pixels) – Mouse detection is done by threshold and selecting the threshold areas. Sometimes due to illumination/shadows, small dark regions can also be picked up. To ignore these regions, we set a minimal area of detection for the mouse. This will depend on the proximity of the camera and size of the animal. To have an idea, open a movie and using the polygon tool, draw a region of interest around the mouse and measure the area for a few frames. The minimal area should be smaller than the average of what you measured. This impacts on the measurements.

Head fraction of body – Depending on the distance of the camera to the base of the apparatus the fraction of the head to the body of the mouse will be bigger or smaller (or if the mouse has a big head!). This is important for determining when the mouse leaves a region or not or when it is exploring. This impacts on the measurements.

Open Field preferences

Default width of the cube (units) – consider only the base of the box when giving this measurement.

Elevated Plus maze Preferences

Width of arms (units) – consider the base of the arm when giving this measurement.

Length of arms (units) – consider the base of the arm when giving this measurement.

Selection smoothing value – We found that the detections on this maze tended to have rough and noise edges. To reduce this, we decided to apply a routine of dilation/erosion to smooth the selected ROI. Normally a value between 2–4 is enough. A higher value can lead to the loss of valid contour properties

of the mouse.

Morris Water maze Preferences

Pool diameter (units) – consider only the base of the pool when giving this measurement.

Distance to pool wall to consider at wall (units) – self-explanatory.

T/Y maze Preferences

Arms width (units) – consider the base of the arms when giving this measurement.

Selection smoothing value – see Elevated Plus Maze preference for explanation.

If you check the default parameters all the above settings will be ignore and the values on Figure 2 will be introduced.

Transforming your video into a TIFF stack

There are several programs that can convert movie files into TIFF stacks. In ImageJ/FIJI one can import a movie using FFMPEG plugin (available from the Updater Manager sites). Despite this, we found several problems in getting this plugin to work consistently so we provide two tools for the processing of movie files into TIFF stacks. Both tools require the file **ffmpeg.exe** in the toolset folder of ImageJ/Fiji. The file ffmpeg.exe is bundled in the zip file that can be download from http://ffmpeg.org/download.html. The macro/toolset folder is normally located under the Fiji.app directory.

To note that the conversion of a movie into a TIFF stack normally leads to a massive increase in the physical size of the TIFF and a massive number of frames, as the whole movie gets decompressed into its individual frames.

Process Video File

This tool uses ffmpeg.exe to convert a movie file into an AVI file that ImageJ can open. After converting the movie file, it automatically opens it and asks you to have a look and determine if you want to delete any frames. Use the slider to move to the starting frame that you want to save and press OK. Next move to the last frame that you want to save and press OK. Finally select where to save the TIFF stack. The tool will now save the TIFF stack and delete the intermediate AVI file. You can now open the TIFF stack and run your analysis.

Batch Process Video Files

This tool performs the same basic function as the Processing Video Tool, but allows the user to select

a folder containing several videos and a destination folder where the TIFF stacks will be saved. The tool will go through all the videos and create the TIFF stacks. You have no option to visualize or trim down the videos.

Open Field

This tool analyzes a movie of a mouse in an open arena configuration. An open image is required. Once selected, a Dialog appears (Figure 3) where one can setup the image processing and analysis. The first option asks for the **Mice Color** against the background. This is important for later steps of determining the threshold and the background color to apply.

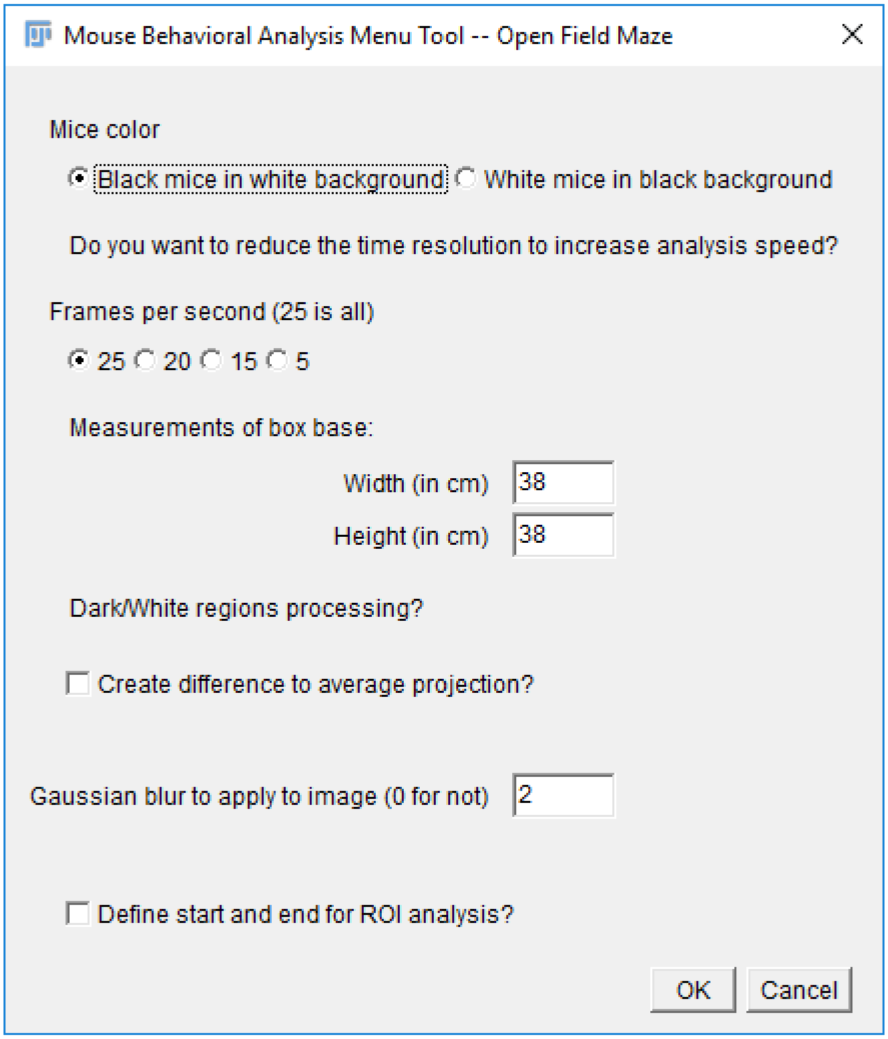

Figure 3. MouBeAT Open Field test dialog

Afterwards one can choose to reduce the time resolution and increase the analysis speed by selecting the **Frames per second** (25 is all). The lower the number of frames per second analyzed the faster the analysis will run but also the less accurate. Next we have the options specific for the Open Field test analysis. These are the dimensions of the box base, namely the **Width** and **Height** in the units select in preferences (these values are automatically filled with the values set in Preferences, but can still be changed here, without affecting the preferences values. Next we have the option to **Create difference to average projection?** This option leads to the creation of an average background image which when subtracted to the original image clears all the background leaving only the mouse. This greatly improves detection of the mouse and our advice is to always use it. The only drawback is

a decrease in the speed of the analysis.

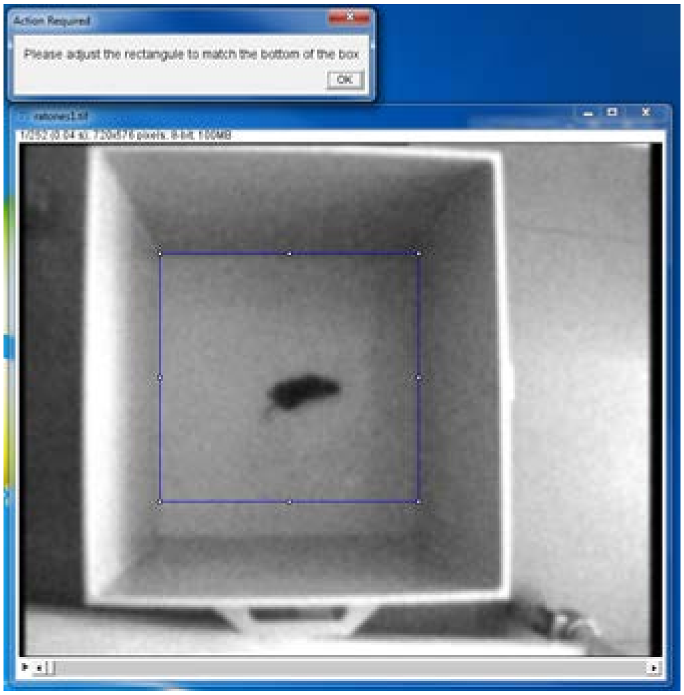

Figure 4: Initial steps in the Open Field Analysis

Next, one can define the size of the sigma value to the **Gaussian blur to apply to image** (a smoothing filter to reduce noise). Last, one can choose to **Define the start and end for ROI analysis**. This allows the user to constrict the analysis of the Regions of Interest (ROI – the mouse) to a specific range of frames instead of the whole movie. Finally press **OK** to proceed with the analysis or **Cancel** to stop here. Let's supposed we thicket both checkboxes and choose a sigma of 2 for the Gaussian blur. The program will first apply the Gaussian blur to the image. After this it will ask the user to adjust a rectangle to the base of the cube (Figure 4). After adjusting the rectangle, it will clear the outside of the selection (an increased selection to account for the borders of the cube). Next, an OK window explaining how to proceed with the processing of the dark/white regions will appear. Press OK. Immediately after, another OK window will ask you to choose the initial frame to start averaging.

Choose the frame by moving the movie slider at the bottom of the movie to the selected frame. Press OK. Another OK window appears asking to select the final frame for averaging. Again select the final frame by moving the slider underneath the movie to the selected frame. Press OK. The program will now create an average projection and make the difference movie of the projection. This helps with eliminating the background of the movie. Another OK window asks you to adjust the threshold. When doing so be sure to check trough the movie that the threshold is ideal. Try to always detect as much as possible of the mouse but without detecting large pieces of shadow or external pieces. An example of two bad thresholds and a good one are shown in Figure 5. Press OK. If selected an OK window will popup asking to select the initial frame to start the Region Of Interest (ROI) analysis. As with the Dark/White regions analysis select the frame by moving the slider of the movie. Press OK. Do the same to select the final frame for ROI analysis. From here the program will make the detections and analyse them. It will write its findings into 2 output files (see output files section). Now you have the choice to create 2 more image based outputs. A **HeatMap** and a **Line Track** from the mouse path. A small dialog with

Figure 5: Two bad examples of thresholds (left and middle panels) and one good one (right panel).

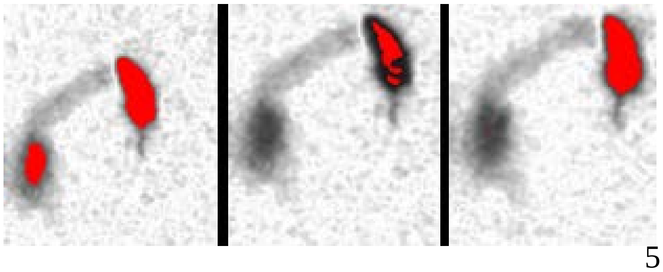

2 checkboxes will appear. Cancel will stop the macro, if you only require the analysis of the ROIs.

Both selections have their individual dialog windows. The HeatMap allows you to choose which type of marker to select. A selection, the detected ROI of the mouse, or the centroid point of the mouse with 1, 4 or 8 pixels size. Finally, you can decide if you want the cube and center region borders to be drawn. The latter two options are also present in the Line Track. The path drawn is always a line between centroid in consecutive frames.

Elevated Plus Maze

This tool allows the analyses of Elevated Plus Mazes. Similar to OF analysis when the option is selected a setup Dialog will appear. This Dialog has several options that are similar to the OF analysis and which were explained there. Here we only explain the specific to the EPM analysis (Figure 6). These refer to the **Measurements of central area / arms of Maze**, where the user inputs the **Width and Height of the arms in units** (it's assumed to be a square the central region) and the **Length of the arms**, also in units. These measurements are important to determine where the mouse is and when the mouse is exploring. The next parameter is also essential for the setup is the **Open arms direction – North – South** (along the Y axis) or **West – East** (along the X axis). After this when you press OK the analysis will proceed in a similar fashion to the OF one. First it will apply a Gaussian blur if so selected. Next, it will ask you to adjust a square to the central area of the Plus Maze. This is important for the measurements. Next, the user is asked to draw a polygon arrow the EP maze. This will be used to Clear Outside the maze. After selecting the range of frames to use to do the average projection, selecting the range of frames to do the ROI analysis and adjusting the threshold the program will detect the mouse, performing the analysis relevant to the maze and ask the user if it wants to create a HeatMap and/or a Line Track.

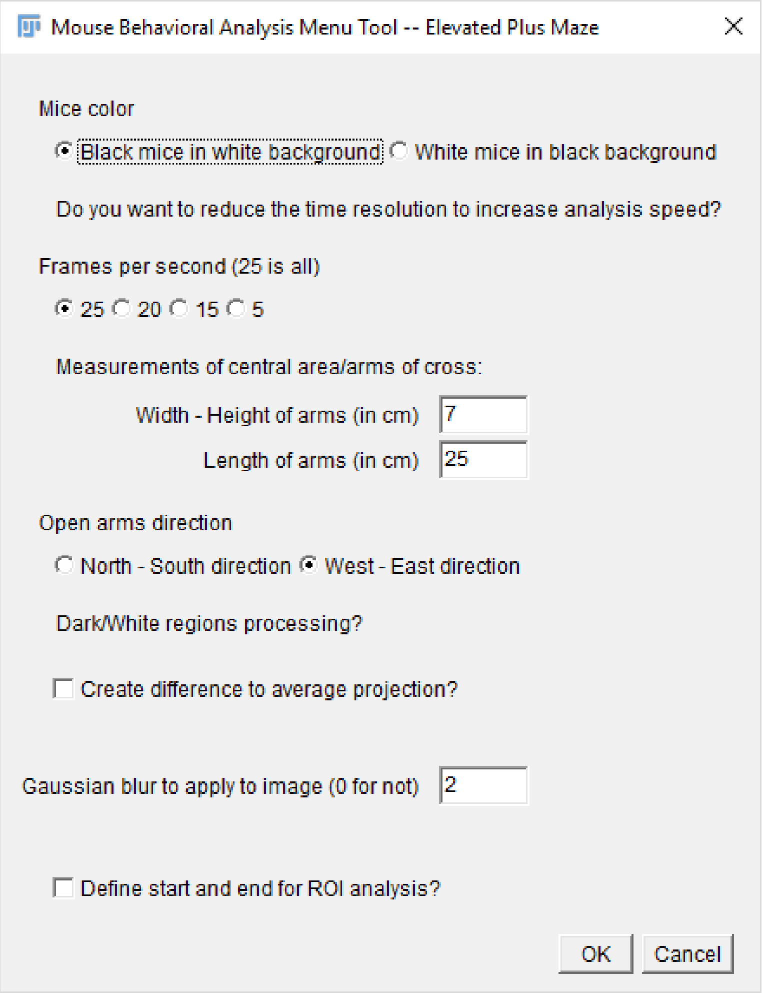

Figure 6: MouBeAT Elevated-Plus Maze dialog

Morris Water Maze

As with previous tools the setup Dialog for this tool is in all similar to the OF setup. The specific parameters here refer to the **diameter of the pool in units,** and an option to r**emove regions of the pool** that will mess up the detections (Figure 7). In this case, we opted for not having the option to perform an average projection treatment because the analysis work fine without it in all our tests, and therefore we did not felt the need to include this option. Once started, the analysis will ask the user to adjust an oval to the base of the pool and another oval to the base of the platform. After this the user will be required to set the initial and final frames for ROI analysis and the threshold. The program will again performed the detection and analysis and afterwards give the option to create the HeatMap and Line Track. In the options for line/heatmap dialog, the user can select **number quadrants and draw border** to visualize four quadrants (Q1, Q2, Q3 and Q4) of the pool.

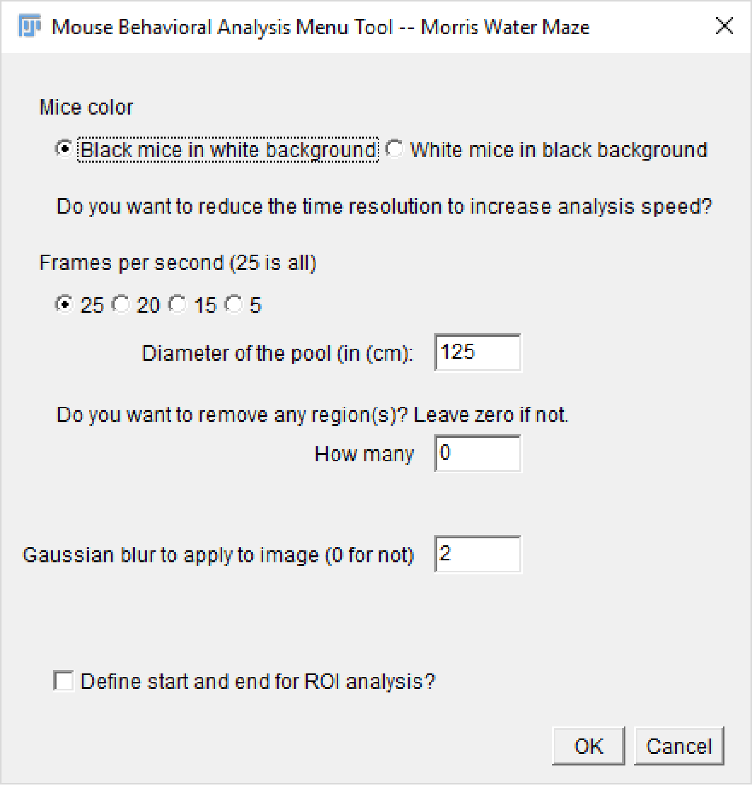

Figure 7: MouBeAT Morris Water Maze dialog

Y-maze

In this maze, the setup Dialog requests the user to enter the **Width of arms** (in units). After starting the procedure the program will apply the Gaussian blur, followed by requesting the user to adjust a triangle to the vertices of the Y arms. This is important to determine in which arm the mouse is located. Next, the user is required to draw a polygon to match the base of the maze. The Tool will then procced to do the average projection difference routine after which the user needs to adjust the threshold. From here the program will perform the analysis and ask the user for the extra images to generate – HeatMap or Line Track – and their relevant options. The user can go to HeatMap or Line Track to visualize the center, left and right arms. As indicated in the upper part of .Spots file, number 1 is used to refer to center arm, 2 to left arm and 3 to right arm.

Open Previous Analysis File

This Tool allows to open a previous analysis file (see Output Files below) and provides a few options (Figure 8) to redo analysis, redo HeatMap and/or Line Track, or just open the movie file and the ROI selections. The user is asked to select a previous analysis file that terminates in .trac and from here, depending on the option selected, the Tool will either open the previous movie file, or if it can´t find it and it is not essential for the selected option, create a blank file movie, using the dimensions present in the .trac file. The options Repeat Full Analysis will take the previous analysis parameters to redo the analysis, including movie processing and ROI detection, while the Repeat selection analysis only, will only analyze the previous ROIs that are saved in a file. Both options will overwrite all the previous output files. Finally, the user can choose to redo the HeatMap and/or Line Track images or just open the previous aimge and ROIs files.

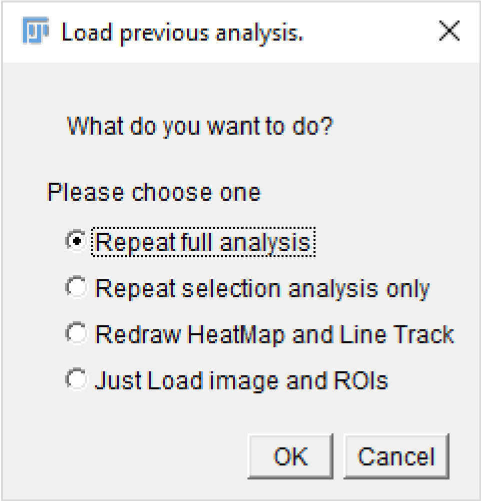

Figure 8: MouBeAT load previous analysis dialog

Ouput Files

All Tools for analysis present in this ToolBox will always output 3 files to the folder of the movie being analyzed. The files will be named with the Title of the movie plus terminations indicating what type of output file they are.

Analysis parameters files

These files are composed by the Title of the movie, together with one of 6 possible terminations and the extension **.trac**.

The possible extensions are:

.cube - for Open Field Maze analysis, eg. NameOfMovie.cube.trac;

.cross - for Elevated Plus Maze analysis, eg. NameOfMovie.cross.trac;

.swim - for Morris Water Maze analysis, eg. NameOfMovie.swim.trac;

.TY - for Y Maze analysis, eg. NameOfMovie.TY.trac;

These files are simple text files that can be opened directly on ImageJ/Fiji or any other text editor where the parameters of the analysis are saved. These files allow a previous analysis to be remade applying the same settings as previously (namely, threshold levels, areas to clear outside regions analyzed

average projection initial and final frames, and ROI analysis initial and final frames). The first line of these files always has the directory of the movie when it was analyzed, the dimensions in X, Y, and T of the movie and the value of sigma applied when of the analysis. The second line has the file name of the movie analyzed. From here onwards, each line starts with the name of the parameters that are referred to in that same line. In addition to the parameters of the analysis, this file also contains the values of the Preferences at the time of analysis. When redoing an analysis to recalculate the program will output to the log window a comparison of the Preferences of the first time you did the analysis, to the current Preference values, and warn if they are different.

Frame by frame result files

These files are ImageJ/Fiji result type of files (normally a CSV file that can be open with ImageJ/Fiji or Excel) where the analysis of the ROI for each frame is output. They are named by adding .**Spots** to the name of the movie and saved to the current folder of the movie with the extension .**xls**.

The user can visualize in these files what the Tool detects frame by frame. The output is organized in columns with each column title describing the output generated, which can be numeric or textual. The number of columns and their contents will obviously vary in between Tools.

Overall result files

These files are ImageJ/Fiji result type of files (normally a CSV file that can be open with ImageJ/ Fiji or Excel) where the global analysis of the ROIs for each specific Tool is calculated. They are named by adding .**Track** to the name of the movie and saved to the current folder of the movie with the extension .**xls**.

These files are organized in rows, each row with a descriptive label and the corresponding value in front. These files basically, make an overall analysis of the **.Spots** files and present a summary of the findings.

ROIs file

These files are ImageJ/Fiji ROI saving files with the extension .zip. They can be opened by ImageJ/Fiji and contain all the ROI detections of an analyses. The files are named by the movie filename added with the word **ROIs**.

Known problems and workarounds

1. The tool fails immediately after the Setup Dialog with an error that a file already exists.

This problem is normally due to a previous analysis that was made but either cancelled or crashed, leaving the output analysis file unclosed. The file is therefore not realeased by ImageJ/Fiji (if the session hasn´t been closed) and ImageJ/Fiji cannot delete this file when it tries to redo the analysis.

This is normally solved by double clicking in the status bar in ImageJ/Fiji or deleting the file in question. To find out the file name, check the Output Files section.

2. The selection for adjusting to the base of the cube/pool/platform does not appear when I´m asked to adjust it.

This is normally due to a refresh/update of the image problem. Normally by just placing the mouse over the image and pretend to drag the selection, it should appear. If not, just select the type of tool that you are supposed to use (this is normally already selected) and drag a new selection. This will work just as well.

3. The processing Video Tool is taking forever. Should I cancel it?

This step can take quite a long time depending on the size of the movie and computer properties. The program will output an exit window with the message that the process has finished. If you want to verify that the program is frozen, check the folder of the movie to see if the files that it is creating our increasing in size or not. If they are not increasing in size the program might be frozen. Also make sure that your ImageJ/Fiji program is configure with enough memory to be able to open an AVI movie without using Virtual Stack (Edit>Option>Memory and Threads).
